# “Illness Calls for Stories”: Care, Communication, and Community in the COVID-19 Patient Narrative

**DOI:** 10.1007/s10912-023-09835-9

**Published:** 2024-02-13

**Authors:** Rosalind Crocker

**Affiliations:** University of Sheffield (School of English), Sheffield, UK

**Keywords:** COVID-19, Community, Social media, Narratives of care, Curative recipes

## Abstract

This creative-critical piece reflects on the practices of recording, communicating, and caring that took place on social media and in digital spaces during the COVID-19 pandemic. Using my own experience of contracting COVID-19 as a starting point, the piece looks at the ways in which epidemics have often been recorded in collaborative ways, with the personal, professional, and familial converging in historical texts that could be used as sources of medical authority. COVID-19 has similarly been immortalized across a variety of forms and by different communities. The piece particularly explores the ways in which collective epidemic experience has been represented online through autopathographical Tweets, TikTok cures, and group chat messages and the future purposes that such collaborative patient narratives can serve.

One evening, I became aware of a dull ache behind my eyes, then a tingling, pin-pricking sensation on my skin, a sign which, for me, usually signals an oncoming cold. I began to feel hot, inexplicably hot for a relatively mild June evening. As I tried to move upstairs, I realized my limbs felt so heavy I could barely stand up. My partner found me later when he came home from work, curled up and unable to face the walk to the kitchen for the painkillers I so desperately wanted. That was day one.

On day two, my headache got worse, and I could not bear to look at light. I took the test, which confirmed my suspicions. On days three and four, I was being sick every half an hour. On day eleven, I tested negative for the first time but still struggled to walk up and down the stairs. Prior to my experience of COVID-19, I am lucky to have had little need to reflect seriously on the experience of being incapacitated with illness. My days in bed, staring at the same wall, gave me time to consider what this would mean. I also began to think about how, if at all, I would want to record this aspect of my pandemic years.

If, as Arthur Frank (1995) notes, “illness calls for stories,” I wonder what stories the last few years have generated. Studying historical patient narratives, I focus largely on the immortalizing influence of such accounts and the ways in which illness has been recorded and later revisited by readers. I am interested in narratives of care, particularly self-care, shared within communities, supplementing or even replacing traditional sources of medical authority. The Wellcome Collection holds numerous examples of such materials; Fig. 1 shows a household recipe book from the seventeenth century, wherein cooking instructions sit alongside homemade plague remedies alongside prescriptions from local practitioners. Compiled in various hands and over multiple generations, the personal, the professional, and the familial converge in such texts. Medical knowledge and experience have always been a currency, but never more so than during epidemics, where the cost of contagious illness is most acutely and visibly experienced as a shared burden.

**Fig. 1:**
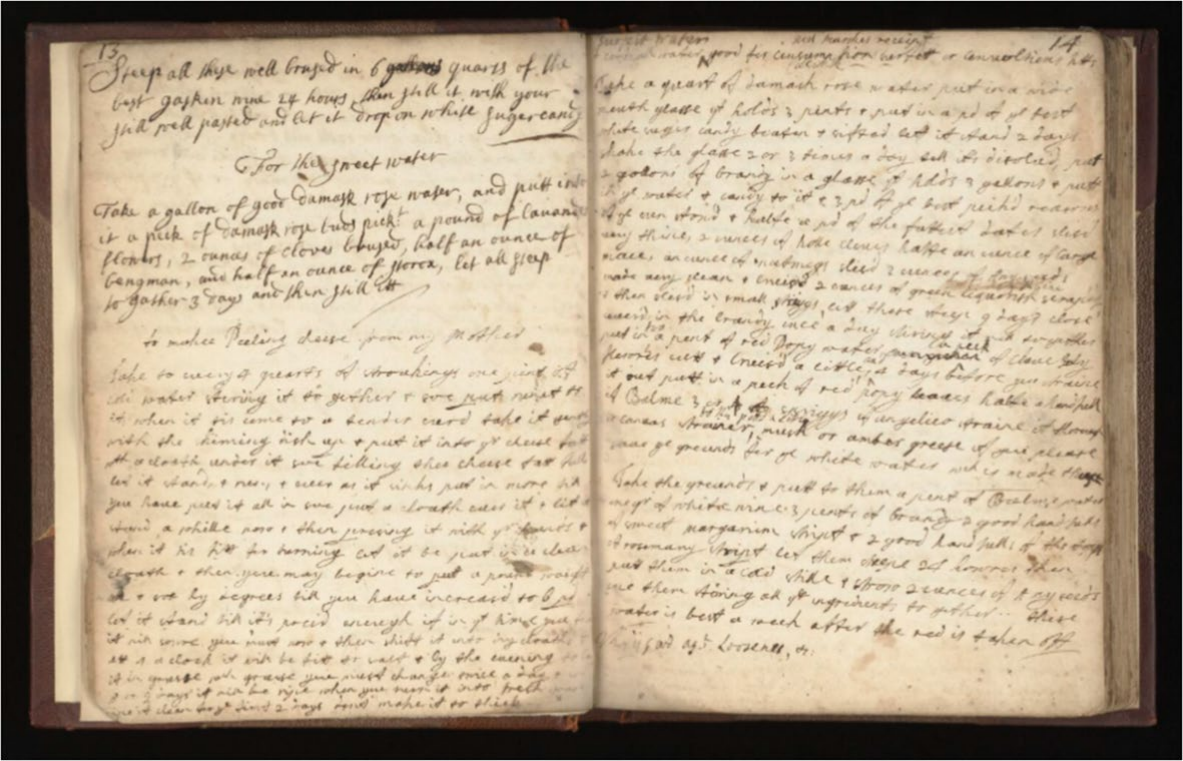
Wellcome Collection. n.d. *Household and Medical Recipes, c.1650-c.1750.* Ref. MS.MSL.2. https://wellcomecollection.org/works/d2ckruhz

Though my research is largely based on contemporary epidemic experience, working with these early modern archival materials sparked a new interest in the various modes and meanings of the “patient narrative.” What forms of knowledge are produced in texts that are both reflective and advisory? How are they communicated and adapted over generations? Anne Stobart looks at the ways in which similar collections of medicinal recipes from the seventeenth century communicated observed facts but also the concerns, opinions, and beliefs of members of the immediate household and wider networks of friends and family (Stobart 2016).

This impulse to record epidemic events collectively has echoes in the twenty-first century. COVID-19 memoirs began emerging in the very earliest throes of the pandemic, but not perhaps as we might immediately recognize them. I found the most immediate and most urgent “patient narratives” through social media, 140-character fragments of the most catastrophic public health emergency in recent decades. With face-to-face communication presenting new threats in terms of viral infection, social media spaces were utilized to record the pandemic experience but also to relay and share information in real time. Significantly, these narratives were both textual and visual, with the experience of hospitalization and treatment, key messages around mask-wearing, and displays of support rendered frequently through emojis, relaying a form of “digital feeling”  ; praying hands, hearts, and rainbows—frequently associated in the United Kingdom with the National Health Service (NHS)—were among the most commonly used (Al-Rawi et al. 2020). Though different in format from traditional patient narratives, in content, these short-form accounts can be read as part of a familiar canon of epidemic textual production. Such accounts offered warnings, sharing the symptoms discovered by lived experience that had not yet found their way into official guidance (Krittanawong et al. 2020). They also offered reassurance in the face of the widespread panic and fear of the unknown that characterized those first few months. Care for others was embedded in such narratives.

Advice for treatment and prevention were also commonplace on social media, evidencing this sense of interpersonal care and responding, perhaps, to a desire to regain personal control in a spiraling global situation. Alongside the advice of medical practitioners and public health officials, the lived experience of COVID also granted a sense of legitimacy to apparent “cures.” Although such interventions could highlight the democratizing potential of social media as utilized during public health events, there is also a blurring here between medical *knowledge* and medical *information*, with personal experience reinterpreted as advice. This melding of anecdotal experience and clinical forms of knowledge continues a historical precedent in times of widespread contagious illness, with community care supplementing the guidance deployed by institutional medicine. Stobart refers to another seventeenth-century medicinal recipe collection, of the aristocratic Clifford family, which attributes its contributions to a variety of figures with institutional and familial titles; doctors sit alongside captains and countesses (Stobart 2016). Utilizing readily available products and designed to be replicated as often as needed, such recipes are largely comprised of foodstuffs like oils, suet, herbs, and other identifiable plants.

In another contemporary parallel, food also played a key role in advice for COVID care offered on social media. Loss of taste was one of the lingering COVID symptoms most frequently addressed in such guidance, with the hashtag #covidtastetest appearing on TikTok 28.1 million times between May 2020 and April 2021 (Michell 2023). Adrianna Grace Michell looks to the “burnt orange” phenomenon, which was first suggested as a cure for COVID taste loss in December 2020, the original video garnering upwards of 20 million views and hundreds of thousands of responses. Originating with TikTok user Kemar Lalor (@toosmxll), the remedy (Fig. 2) comprises of burning, peeling, and eating an orange with brown sugar; as Michell notes, the recipe is attributed to Lalor’s grandmother, enacting an intergenerational call to authority. In a voiceover, Lalor highlights the appeal of anecdotal, rather than clinical, curative guidance—“I’m no scientist but it does work, I’m telling you” (Lalor 2020).

**Fig. 2:**
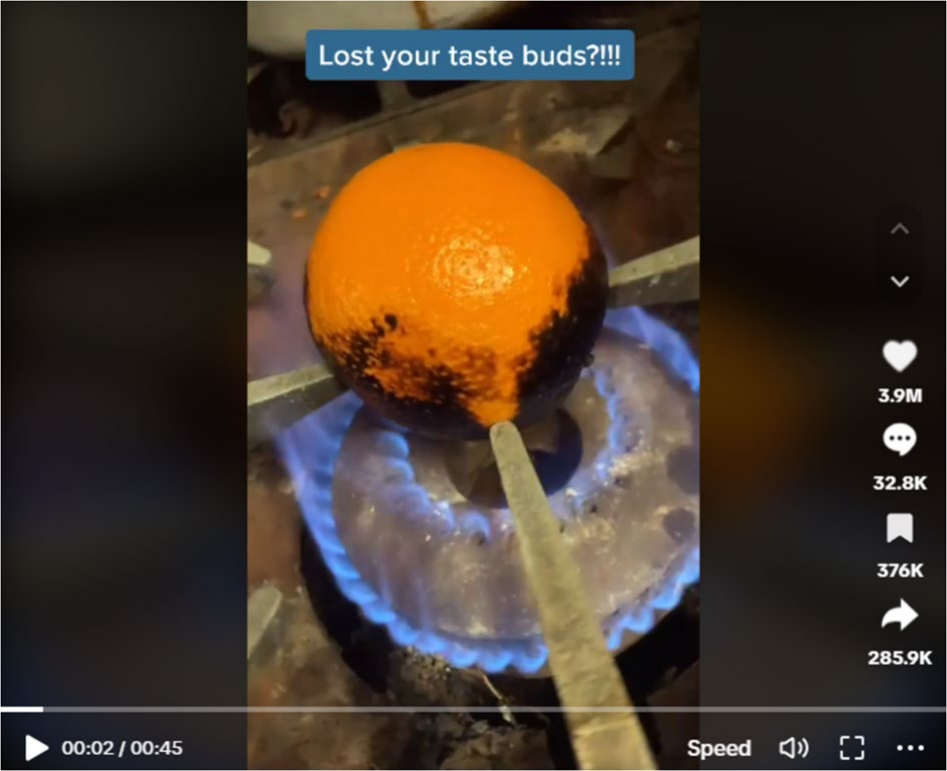
Lalor, Kemar (@toosmxll). 2020. “How to get taste buds back!!! #howto #tastebud #lifehacks #health #fyp #remedy.” TikTok, December 9, 2020. Accessed November 3, 2023. https://www.tiktok.com/@toosmxll/video/6904461007586987265?lang=en

As much a social media trend as a source of medical guidance, these videos, as well as similar short-form online narratives, have become more difficult to revisit as the COVID years have worn on. Like most timeline-based social media platforms, TikTok organizes the videos presented to viewers by algorithmic relevance, and as COVID has slipped out of immediate public perception, so too have the narratives and community-led guidance responding to it. In preparing this piece, I scan through my phone rather than my bookshelves, aware of the ephemerality of these sources in comparison with the concrete materiality of the plague recipe book. I look for Lalor’s video, needing to filter my search results multiple times before it appears; once omnipresent, the most recent “burnt orange” COVID taste test was posted on the app three months ago.

What might an archive of COVID-19 patient narratives look like in this context, where sources can so quickly become much harder to locate? Much of my recollection derives from messages between friends, urgent phone calls, and ominous notifications from the NHS app. I search an old group chat (Fig. 3), looking for a few keywords. *Isolate. Positive*. Ping. When I find something relevant, I screenshot it. This is textual production, but in a far more fleeting sense, a material record of the pandemic, but mediated through a digital interface, a screen. Such barriers serve both to protect and isolate, and I am reminded, looking at my pandemic memories through touch-screen glass, of Perspex partitions in supermarkets and reunions through hospital windows.

**Fig. 3:**
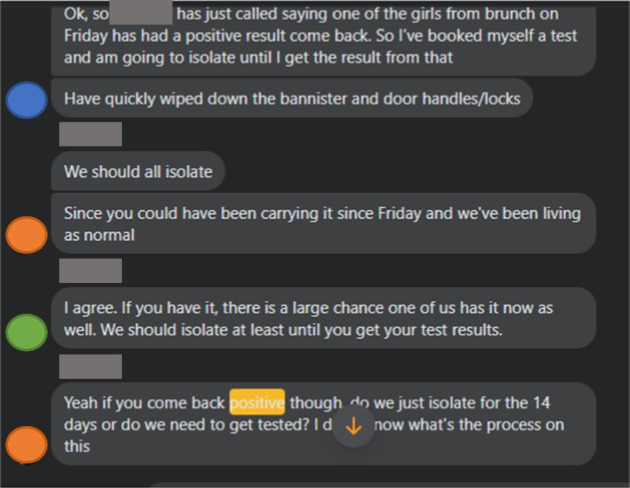
Screenshot from the author's group chat, anonymized and shared with permission (2020)

Such narratives also, however, evidence a necessarily interpersonal aspect of the pandemic experience, a dependence on one another to keep ourselves safe. Where official guidance faltered, decisions about care and preventative action were made in these digital spaces. Curative recipes, relying on generational, experiential knowledge and readily available materials, supplemented clinical care. On social media and through instant messaging, we checked in on symptoms, changed plans, liaised about using communal rooms, and created a collaborative narrative of our pandemic years. When my parents contracted COVID months after my illness, I revisited these messages so that I was able to track their symptoms, day by day, in line with my own experiences and those of friends, warning them of what might be to come, reassuring them of what was soon to pass. My patient narrative, collected through bemoaning Tweets and half-lucid messages, began to serve a new purpose, not only immortalizing or coordinating but also informing and comforting.

Patient narratives have always functioned in a number of ways, but shared widely through social media, COVID patient narratives might present even more obvious capacity for care and opportunity for community-building. Felicity Callard and Elisa Perego (2021) have described Long COVID in particular as a “patient-made” illness, with online communities exchanging experiences and campaigning to raise awareness before the condition was afforded full clinical recognition. In this way, the patient narrative presents a call to futurity, of possibility, as well as an act of remembrance.

As I write in 2023, around the third anniversary of the first national lockdown in the United Kingdom, these multiple temporalities of the patient narrative are all the more apparent. The National COVID Memorial Wall (Fig. 4), currently comprising more than 220,000 hand-painted hearts to represent the UK’s COVID death toll, continues to be updated. Reminiscent of the hearts that adorned social media “walls,” this symbolic, visual rendering of the scale of loss experienced through the pandemic is a similarly collaborative and community-led enterprise. Social media and instant messaging have ensured that the immediate experience of COVID-19 continues to be well-recorded; it is important now that these accounts are not allowed to simply disappear down the timeline but endure as a public, communal narrative of the pandemic.

**Fig. 4:**
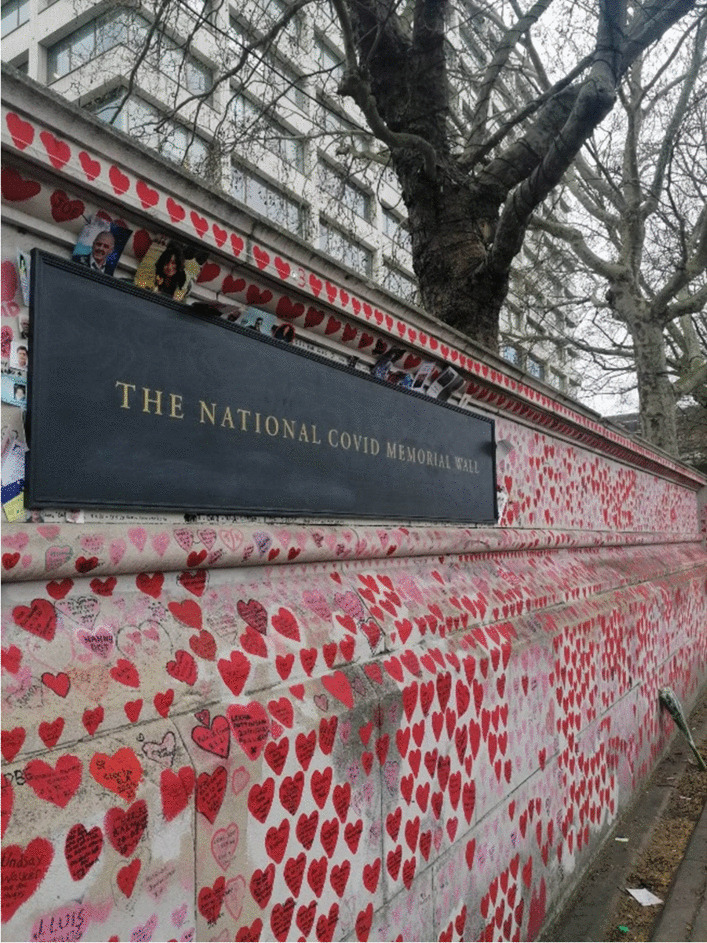
The National COVID Memorial Wall, London. Author's own photograph (2022)
